# Supplementary material for: Seed Priming with Single-Walled Carbon Nanotubes Grafted with Pluronic P85 Preserves the Functional and Structural Characteristics of Pea Plants
Source: Nanomaterials (Basel). 2023 Apr 11;13(8):1332. doi: 10.3390/nano13081332 (PMC10143637; doi:10.3390/nano13081332)
Supplement: Supplementary file 1 [file nanomaterials-13-01332-s001.zip › nanomaterials-2311334-supplementary.pdf]

Seed Priming with Single-Walled Carbon Nanotubes Grafted with Pluronic P85 Preserves the Functional and Structural Characteristics of Pea Plants

Sashka Krumova [1], Asya Petrova [2], Nia Petrova [1,3], Svetozar Stoichev [1], Daniel Ilkov [2], Tsonko Tsonev [1], Petar Petrov [4], Dimitrina Koleva [5] and Violeta Velikova [1,2,*]

[1] Institute of Biophysics and Biomedical Engineering, Bulgarian Academy of Sciences, "Acad. G. Bonchev" Str., Bl. 21, 1113 Sofia, Bulgaria; sashka.b.krumova@gmail.com (S.K.); zlatkova.nia@gmail.com (N.P.); svetlio85@abv.bg (S.S.); tsonev@gmail.com (T.T.)

[2] Institute of Plant Physiology and Genetics, Bulgarian Academy of Sciences, "Acad. G. Bonchev" Str., Bl. 21, 1113 Sofia, Bulgaria; petrova_assya@abv.bg (A.P.); daniel_ilkov@yahoo.com (D.I.)

[3] Institute of Plant Biology, Biological Research Centre, Temesváry krt. 62, 6726 Szeged, Hungary

[4] Institute of Polymers, Bulgarian Academy of Sciences, "Acad. G. Bonchev" Str., Bl. 103, 1113 Sofia, Bulgaria; ppetrov@polymer.bas.bg

[5] Faculty of Biology, Sofia University, "St. Kliment Ohridsky", 1000 Sofia, Bulgaria; koleva@biofac.uni-sofia.bg

* Correspondence: violet@bio21.bas.bg

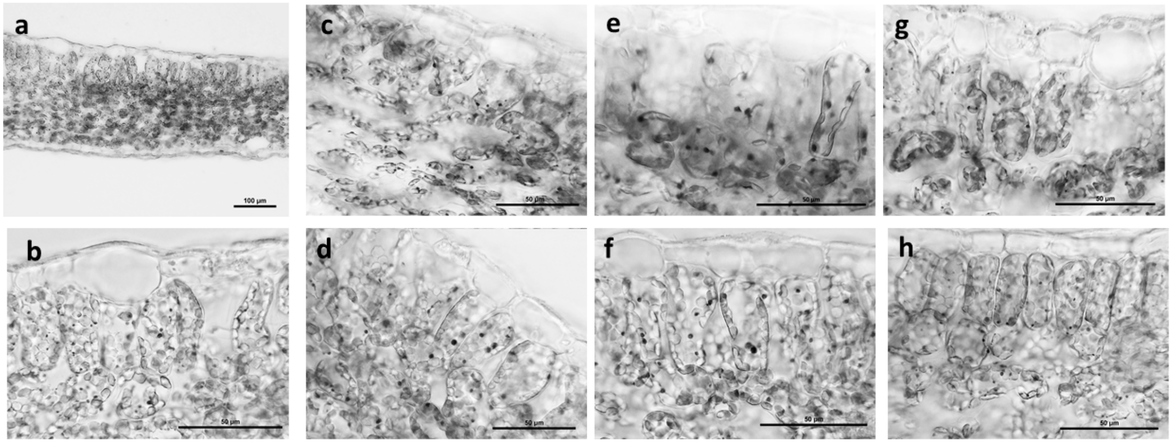

Figure S1. Leaf sections obtained for 14-day-old plants. Images are shown for hydro- (a,b), 50, 100 and 300 mg/L P85-SWCNT- (c,e,g, respectively) and 5, 10 and 30 g/L P85- (d,f,h, respectively) primed seeds. Scale bar 100 μm for panel (a) and 50 μm for panels (b-h).
